# Viral infections of pigs used for medical education. A Japanese experience ^1^

**DOI:** 10.1590/s0102-865020200080000008

**Published:** 2020-09-21

**Authors:** Eiji Kobayashi

**Affiliations:** I MD, PhD, Department of Organ Fabrication, Keio University School of Medicine, Shinjuku-ku, Tokyo, Japan.

**Keywords:** Virus Diseases, Swine

## Abstract

Infectious viruses pose a threat to all living organisms, including humans, and can cause significant morbidity. Previous experience with pigs in medical education and research, rather than in domestic control settings, has led to a unique perspective on viral infections in swine. In this article, common porcine infectious diseases have been listed, based mainly on the authors’ experience thus far. For example, young domestic pigs that were used in surgical training and infected with hepatitis E were subjected to quarantine and isolation treatment, and attempts were made to develop a DNA vaccine for swine influenza arising from swine-to-human transmission. More recent research has focused on preventing infection by the African swine virus, a current threat. We hope that this article of porcine infectious diseases identified at the School of Medicine will help develop a breakthrough with regard to coronavirus disease.

## Introduction

Throughout my research career, I have used laboratory pigs in the context of medical surgery education and preclinical research at medical universities^1^ . In particular, my research career has focused on the creation of transplantable human organs. I have studied the potential use of living pigs as in vivo bioreactors for the differentiation and maturation of human stem cells^2^ , and my research team has tested this concept in small animals and reported our findings in an earlier publication^3^ .

In rodents, severe combined immunodeficient (SCID) technology causes immunodeficiency and enables the development of xenobiological models involving human cells. SCID pigs are also available, and those not subjected to strict infection control may still survive under specialized conditions, such as those provided by definitive pathogen-free (DPF) management^4 , 5^ . Because SCID pigs are difficult to breed, I have developed operational SCID pig models that can be produced using surgical techniques at general animal experimental facilities^6^ . In Japanese laboratory facilities, these immunodeficient pigs have become extremely useful in studies that aim to verify the safety and efficacy of human regenerative medicine products^7^ .

The above-mentioned studies require the long-term maintenance of human-derived cells in living pigs. Therefore, measures are needed to prevent the transmission of zoonotic diseases between pigs and humans. In a chimeric pig, a virus that normally infects only pigs might attain the ability to infect humans, or vice versa. Therefore, in addition to DPF management, we have actively explored the prevention and treatment of viruses that target pigs.

In this article, first, the commonly reported infectious diseases of pigs have been summarized in Table 1 . Information on the transmission of these infectious diseases that affect domesticated pigs and relevant countermeasures have been described in other reviews^8^ . Worldwide, domestic pigs are being used in areas such as transplant research and surgical training as well as in the development of novel medical devices. At our School of Medicine, it is necessary to have adequate knowledge in the field of animal husbandry as it relates to these abovementioned fields.

**Table 1:** Types of infectious disease of pigs and reports of human infections.

Infectious Diseases of Swine*	Reports of Human Infections
African swine fever (ASF)	None
Aujeszky’s disease	Reported
Brachyspirosis	None
Pig cholera (CSF)	None
Cryptosporidiosis	Reported
Foot-and-mouth disease (FMD)	None
Hepatitis E virus (HEV)	Reported
Lawsonia intracellularis	None
Mycobacterium avium	None
Mycoplasma hyopneumoniae (M.hyo)	Reported
Parvovirus infectious disease/Porcine parvovirus (PPV)	None
Pasteurella infection	None
Porcine circovirus type 2 (PCV2)	None
Swine epidemic diarrhea	None
Porcine reproductive and respiratory syndrome (PRRS)	None
Porcine Respiratory Coronavirus (PRCV)	None
Rotavirus	Reported
Salmonellosis	Reported
Brachyspira hyodysenteriae	None
Swine dysentery	None
Pig delta coronavirus	None
Swine flu (SIV)	None
Swine vesicular disease (SVD)	None
Toxoplasmosis	Reported
Infectious gastroenteritis	None
Trichinosis	Reported

This article introduces the measures used to combat viral infections in pigs, describes some relevant experiences, and recommends strategies for the future from the perspective of medical research^9^ .

## Investigation of hepatitis E virus contamination in domestic and experimental pigs and isolation breeding trials

I have long participated in surgical education at medical universities^1^ . Three decades ago in Japan, wild dogs captured at public health centers were used for surgical experiments and training purposes. In 2000, I launched a publicly funded educational program that uses domestically bred pigs for medical training at a medical university.

Approximately two decades ago, the hepatitis E virus (HEV) was detected in commercially available swine liver in Japan, and many patients with HEV in a single region reported having eaten raw swine liver before the onset of the disease. The pathogenicity of HEV in domestically bred pigs is low. In pigs that were experimentally infected with a swine-derived strain of HEV (type III), swollen hepatic hilar and mesenteric lymph nodes were considered gross lesions, and lymphocyte-plasma cell hepatitis and hepatocellular necrosis were recognized as histological lesions. Although clinical symptoms and elevated levels of liver-derived enzymes such as alanine aminotransferase were not observed, viral gene expression was detected in the liver, bile, feces, and serum of young pigs for more than 2–3 weeks. A 2003 report on the status of livestock in Japan reported the following information: 1) 90% of pigs aged 5–6 months were previously found to harbor antibodies to HEV, indicating a previous infection, and 2) HEV was not detected in 6-month-old (usually shipped) pigs. In other words, pigs that were shipped for meat processing were deemed virus-free^10^ . At medical universities, however, 3–4-month-old domestically bred pigs were often used for research and training.

During that time period, we were investigating the HEV statuses of pigs owned by farmers who were affiliated with medical universities and with a company that sold miniature pigs for experimental use^11^ . Our research revealed that approximately 1% (1/84), 6% (1/16), and 38% (20/52) of the domestic pigs from farms A, B, and C, respectively, had detectable HEV RNA levels. The 412-nucleotide sequence of ORF2 in the 22 HEV isolates recovered from viremic pigs that shared 89.8–100% similarity and could be segregated into three clusters within genotype 3. Although HEV RNA was detected in 1 pig from farm A, the HEV isolate was nearly 100% identical to the isolates recovered from farm C pigs, and the serum samples from all 84 pigs at farm A had negative anti-HEV antibody titers. These results suggested that farm A was free from HEV infection. The viremic pig from farm A had been raised in a barn at our center for 1 month before serum sampling, and HEV-viremic farm C pigs had been reared for several days in the same barn approximately 3 months earlier. Most likely, the pig from farm A had been infected with HEV in our barn. An additional 38 miniature pigs from farms D and E were negative for both anti-HEV antibodies and HEV RNA. To further investigate the prevalence of HEV infection, pigs that were raised at four swine farms (A, C, D, and E) were subjected to anti-HEV testing. Although 96 (86%) of the 112 pigs from farm C had positive antibody titers, none of the 48 pigs from farm A or the 138 miniature pigs from farms D and E had positive results. Taken together, these data suggested that some Japanese farms were not contaminated with HEV and that virus-free environments could be created by implementing appropriate isolation measures.

Based on the above findings, farmers and commercial buyers of miniature pigs for experimental use decided to first evaluate the degree of HEV contamination in each pig and then place and manage the virus-free animals in a dedicated facility. Furthermore, newborns from HEV-contaminated farms, which were considered to have acquired maternal immunity, were weaned and isolated in a newly constructed swine barn in collaboration with a company that bred and sold specific pathogen-free pigs. However, this effort did not produce satisfactory results (unpublished data). These cumulative observations demonstrate the limitations of isolation measures and indicate that when aiming to control the spread of a viral infection, organisms may be transmitted under some circumstances, despite the subclinical nature of the infection.

Several studies have reported that in clinical practice, hepatitis E can be transmitted to patients via blood transfusions. These studies also found that subclinical HEV infection tends to persist in patients who have undergone organ transplantation, possibly due to the postoperative use of immunosuppressant agents^12^ . Other studies have identified pig and boar meat as sources of HEV infections. Taken together, the data emphasize the importance of HEV screening for early detection and treatment^13^ .

## Influenza H1N1 virus infection in pigs and humans and DNA vaccine development

The term “swine flu” is applied to any type of influenza that affects pigs. Various genera of the Orthomyxoviridae family have been identified, including influenza C and influenza A viruses; the latter genus includes H1N1, H1N2, H2N1, H3N1, H3N2, and H2N3, among others. In 2009, the first case of pig-to-human viral transmission was reported. However, this case was attributed to a combination of three different viruses^14^ . Six genes in this virus bore similarities to genes in the N1N2 virus, which has spread among pigs since the early 2000s. Although the H1N1 influenza spread to more than 200 countries and regions worldwide in 2009 and resulted in 18,000 human deaths, the H1N1 virus did not spread to Japan. However, a new swine influenza strain, named “G4”, was recently reported to have originated from the H1N1 swine influenza virus, which was genetically prevalent in 2009. Additionally, we are constantly being urged to be cautious about possible pig-to-human transmission^15^ .

I have conducted naked DNA vaccine studies in a mouse model^16^ . Immunization with naked DNA involves introducing a target protein into a living body. Part of the protein is encoded by DNA, and thus the target protein can be both expressed by and presented on antigen-presenting cells. This method has attracted considerable attention because it is considered safer than conventional vaccination, which achieves immunization by introducing heterologous proteins. In 2006, a target gene was successfully introduced into a pig liver using the hydrodynamic method for the first time^17^ . Concurrently, at the University of Pennsylvania, Weiner et al. explored the development of DNA vaccines against diseases such as dengue fever^18^ and attempted to develop vaccines against H1N1 that would be delivered to pigs via electroporation^19^ . However, porcine DNA vaccine research was interrupted because the influenza antibodies present in immune pigs that had been vaccinated made it difficult to determine whether natural infection was occurring. The emergence of a virus that non-discriminately attacks humans and pigs remains an important issue. Vaccines and treatments should be developed in collaboration with people in the agricultural industry who handle pigs and medical personnel who treats humans. The concept of disrupted stock raising will be further explained in the next section on swine fever.

## Detecting the threat of swine fever in Japan and determining measures to overcome this infection

“Swine fever” describes sporadically occurring classical swine fever (CSF) in Japan, as well as the deadlier African swine fever (ASF) that has not yet reached Japan.

CSF is a heat-borne disease in pigs and boars that is associated with high infectivity and mortality rates^20^ . The disease is caused by flavivirus infection, and infected pigs excrete the virus in saliva, tears, and manure and spread infection via contact with other pigs and contaminated items. CSF has been designated as a livestock infectious disease under the Livestock Infectious Diseases Prevention Act because of the enormous effects of outbreaks on the livestock industry. An outbreak of CSF was confirmed in Gifu Prefecture in 2018, and the outbreak then spread to the Aichi, Nagano, Shiga, Osaka, Mie, and Saitama prefectures in 2019. Although pigs that tested positive for CSF were slaughtered, the Ministry of Agriculture, Forestry, and Fisheries advised caution regarding the vaccination of domestic pigs and recognized that it would take time to return to a “clean country.” However, the continued spread of infection in wild boars and other animals has led to the use of vaccines based on attenuated poisoning.

ASF is caused by a virus in the Asfivirus genus and Asfarviridae family. This double-stranded DNA virus differs from the CSF virus. Like CSF, the ASF virus targets pigs and wild boars. However, the mortality rate associated with ASF is extremely high. Japan is not currently contaminated with ASF. Still, ASF virus infections are spreading throughout Asia, and no effective vaccines are available. Vaccine development is actively ongoing at present^21^ , although it is not at a stage where it can be put to practical use. Therefore, policies are being enhanced to prevent the import of raw pigs from contaminated countries. In March 2020, a revised law was enacted to strengthen the penalties for importing illegal meat products. I emphasize that isolation with the aim of preventing infection is important for livestock policy. However, the isolation of infected pigs is associated with economic losses, and has led to further questioning of the importance of preventative measures such as vaccines.

## Conclusions

Many infective viruses have affected humans in the modern era, including the global Spanish Flu pandemic of 1918–19, the ebolavirus that has spread mainly in Africa since 1976, and severe acute respiratory syndrome (SARS), which spread mostly in China during 2002–03. With this paper, I aim to introduce the HEV, H1N1 virus, and swine fever-causing viruses (CSF, ASF) as examples of viruses that attack pigs. Additionally, I have described swine-borne virus infections and measures to combat these diseases. The knowledge presented herein was acquired through medical research involving pigs. This area of research has progressed extensively in recent years, as pigs have been identified as potential xenotransplant organ donors for humans. The studies of swine-borne virus infections introduced in this paper represent an important issue in the field of xenotransplantation research. In addition to isolation measures, the fight against swine-borne virus infections in humans will require the rapid development of both vaccines and viable treatments.

## Footnotes

1 Research performed at Department of Organ Fabrication, Keio University School of Medicine, Tokyo, Japan.
